# Effects of Childhood Trauma and Alcoholism History on Neuropsychological Performance in Adults with HIV Infection: An Initial Study

**DOI:** 10.17303/jaid.2017.3.101

**Published:** 2017-07-02

**Authors:** Stephanie A Sassoon, Anne Pascale Le Berre, Rosemary Fama, Priya Asok, Cheshire Hardcastle, Weiwei Chu, Adolf Pfefferbaum, Edith V Sullivan

**Affiliations:** 1 Neuroscience Program, SRI International, Menlo Park, CA, USA; 2 Department of Psychiatry and Behavioral Sciences, Stanford University School of Medicine, Stanford, CA, USA; 3 University of Manchester, Manchester, UK

**Keywords:** Trauma, Alcoholism, HIV, Cognition, Memory, Comorbidities

## Abstract

**Background:**

Childhood trauma carries heightened risk for neuropsychological impairment and is a frequent concomitant of HIV infection (H) and alcoholism (Alc). Little is known about compounded effects of childhood trauma and these diseases on cognitive and motor functioning. We queried the relation between childhood trauma history (experiencing at least 1 of 13 specified traumas before age 18) and cognitive and motor performance in HIV infection with and without lifetime alcoholism.

**Methods:**

Relations between childhood trauma history (Tr) and four performance domains (episodic memory, information processing speed, executive function, and fine motor function) were examined via ANCOVAs covarying for age and education in four HIV groups: 21 H+Alc+Tr, 19 H+Alc, 19 H+Tr, and 25 HComp (H comparison group without Tr or Alc).

**Results:**

H+Tr, irrespective of Alc, performed poorly on the episodic memory domain. Specifically, immediate and delayed verbal recall, and immediate visual recall were affected in those with HIV and history of childhood trauma with or without alcoholism history. By contrast, H+Alc+Tr performed faster than H+Alc or H+Tr in information processing speed.

**Conclusion:**

The findings of poorer episodic memory in HIV infection with childhood trauma history corroborates previous reports and now extends findings to H+Alc+Tr trimorbidity. The novel interaction of alcoholism and trauma in HIV infection suggests that information processing speed is slowed with trauma history or alcoholism history alone in HIV but not with HIV+Alc+Tr trimorbidity, possibly reflecting greater impulsivity and hyperarousal in multiply-affected individuals.

## Introduction

Traumatic experiences in childhood carry significant risk for mental, physical, and behavioral problems later in life, and the personal, familial, and societal costs are substantial [1]. A recent literature review reported that the incidence of sexual victimization of American adolescents was upwards of 28%, nearly 19% of adolescents experienced physical abuse, and rates of assault ranged as high as 71% [2]. Early childhood trauma is associated with substance dependence including alcohol dependence and psychiatric disorders such as depression and posttraumatic stress disorder (PTSD) [3,4]. Moreover, early childhood adversity is associated with heightened risk-taking behaviors that increase the likelihood of acquiring serious diseases such as HIV infection [3,5].

Over the past decade, a vast literature has focused on relations between trauma and cognition. Some studies have focused on cognitive sequelae of life trauma in PTSD, such as in veterans cohorts, whereas others have explored impact of childhood trauma on cognition in adults. Irrespective of cohort type, trauma has been associated with smaller hippocampal volume and poorer memory performance [6,7]. Memory impairments in those who experienced trauma include deficits in acquisition, free and cued recall, and recognition memory [8]. Both verbal and visual immediate and delayed recall have consistently been found to be affected in adults with trauma exposure history or with PTSD [9–12].

Executive functioning deficits are frequently observed in adults and adolescents who have experienced early life adversity, particularly abuse and neglect [11,13,14]. Impairment in information processing speed has been noted in PTSD [12]. Despite small sample sizes, a study comparing past month trauma with acute PTSD, past month trauma without PTSD, and a non-trauma comparison group found no difference in information processing speed (assessed by Trail Making Part A); however, the acute PTSD group demonstrated impaired executive functioning (assessed by Trail Making Part B and Stroop Interference) compared with the trauma without PTSD group [9]. De Bellis and colleagues compared neglected adolescents with PTSD, neglected adolescents without PTSD, and healthy controls and found that, after controlling for IQ, the groups with neglect history performed poorer on tests measuring verbal and visual memory, those with PTSD performed similarly to neglected adolescents without PTSD and showed executive function impairment relative to controls; no group differences were found in fine motor function [15]. A later study found similar absence of differences in fine motor function in adolescents with and without maltreatment history [16].

Childhood trauma including abuse, maltreatment, and family adversity are directly and indirectly associated with alcohol problems in adulthood [17–19]. Childhood trauma is associated with an earlier onset of alcohol use [20,21], a more severe clinical profile of alcohol dependence [22], and poorer response to alcoholism treatment [22]. Additionally, it has been found that within the first year post-treatment, alcoholics who experienced abuse are prone to earlier relapse than alcoholics without abuse history [23]. Cognitive sequelae of alcoholism involve episodic memory, information processing speed, attention/executive functions, gait and balance, and visuospatial ability, even after substantial sobriety [24–27]; for review, [28]. In a study examining cognitive function in alcoholism and PTSD, alcohol abuse history was found to be associated with poorer visual memory, whereas PTSD was associated with poorer information processing speed, memory, and attention [29]. It has been reported that alcoholics with a history of childhood trauma may drink to alleviate post-trauma sequelae, such as anxiety and depression symptoms, trauma-related intrusions, and unprocessed memories of the trauma, and heavy drinking can lead to risky and impulsive sexual behaviors [22,30] affording a pathway through which HIV infection may occur.

A recent literature review reported that trauma has been experienced by up to 90% of HIV-infected individuals [31] depending on clinical sample and definition of trauma, and rates of heavy drinking in those with HIV infection are almost twice that of the general population [32]; thus, the co-occurrences of trauma and HIV infection, and alcoholism and HIV infection, is common. Trauma [31,33] and alcoholism [34] are each related to poorer adherence to HIV medications and poorer immunologic outcomes.

HIV-associated cognitive sequelae include problems with attention and executive function, psychomotor speed, and memory [35,36]. Little research has been conducted on cognitive function in comorbid childhood trauma and HIV infection.

Two recent studies by Spies and colleagues found that cognitive functions such as processing speed, attention, executive function, motor skills, and learning are hindered in HIV infected women with childhood trauma history; verbal and visual memory were not examined [37,38]. Reduced psychomotor/processing speed, measured by Trail Making Test Part A and Wechsler Adult Intelligence Scale III Digit Symbol-Coding and Symbol Search, was evident in HIV-positive and HIV-negative adults with exposure to ≥ 3 childhood traumatic events (e.g., abuse, neglect, bullying, family conflict) compared to HIV-negative adults with < 3 childhood adversities [39]. Explicit memory, psychomotor impairment, immediate episodic memory, and fine motor speed have been documented in comorbid alcoholism and HIV infection [40–42], and synergistic effects of HIV infection and current heavy drinking were found on motor and visuomotor speed [43]. It is unknown; however, whether individuals with both alcoholism and a history of childhood trauma in HIV infection carry increased risk of impairment on cognitive processes notably affected in these conditions.

In summary, trauma, alcoholism, and HIV infection, individually and together, can have profound impact on social functioning in adulthood and are associated with academic underachievement, unemployment, legal problems, interpersonal problems, internalizing/externalizing disorders, poor emotional regulation, and poorer quality of life. Although history of childhood trauma, alcoholism, and HIV infection can be prevalent as a “trimorbidity” and each condition has been found to be associated with poor cognitive function, the differential relations of all three conditions with cognitive function, to our knowledge, have not been examined. In this study, we queried the relations of childhood trauma, alcohol dependence, and cognitive function in 84 adults with HIV infection. We employed a four group design [trauma history (yes/no) and alcohol dependence history (yes/no)] to test the independent and interactive contributions of trauma and alcoholism history in HIV infection to four domains of neuropsychological performance: executive functions, information processing speed, episodic memory, and fine motor function. Our overarching hypothesis was that childhood trauma history status and alcoholism history status in HIV infection would yield significant interactions on the four performance domains, revealing compounded detrimental effects of these conditions in HIV infection. Additionally, we sought to identify contributions to performance from cognitive and motor processes to determine what underlying constructs may be related to interactive and independent contributions of alcoholism and trauma history in HIV infection. This is the first study of all three conditions together and could expand knowledge regarding the nexus of physical, psychological, and cognitive functioning in individuals with this “trimorbidity”.

## Method

### Participants

All 84 participants were seropositive for HIV infection and were divided into four groups based on childhood trauma and alcohol status: (1) H+Alc+Tr: 21 HIV-positive participants who met DSM-IV-TR criteria for lifetime alcohol dependence and had a history of childhood trauma; (2) H+Alc: 19 HIV-positive participants who met DSM-IV-TR criteria for lifetime alcohol dependence (one subject met alcohol abuse criteria) without history of childhood trauma; (3) H+Tr: 19 HIV-positive participants with history of childhood trauma who never met criteria for alcohol dependence and who never consumed >6 drinks per day for men or >4 for women; and (4) HComp: an HIV-only comparison group comprising 25 HIV-positive participants who neither had history of childhood trauma nor met DSM-IV-TR criteria for lifetime alcohol dependence. Participants were part of an ongoing longitudinal study on the effects of HIV infection and alcohol on brain structure and function. All were recruited from the greater San Francisco Bay area through referrals from community treatment centers or medical clinics, presentations about the study at transitional sober living environments or support group meetings, response to flyers, outreach at community functions such as AIDS Walk, or through word of mouth. The study was reviewed and approved by the Institutional Review Boards of SRI International, Stanford University School of Medicine, and Santa Clara Valley Medical Center, and all volunteers provided written informed consent for study participation.

### Procedure

Participants were screened to exclude history of bipolar disorder or schizophrenia, injury potentially affecting the central nervous system, loss of consciousness greater than 30 minutes, history of chemotherapy, or factors precluding MR scanning. Those eligible by screen were invited to our laboratory for a more detailed medical and psychiatric assessment.

During the medical assessment, participants were interviewed by trained and calibrated clinical researchers about their medical history and medication use. All participants were HIV-positive; blood samples confirmed HIV status via HIV antibody test. CD4 T-lymphocyte count with lower counts reflecting greater immunocompromise, and plasma viral load (log10 transformed) with higher counts reflecting higher concentration of disease were also obtained; Hepatitis C status was also tested. Those with neurological disease (e.g., history of stroke, multiple sclerosis, epilepsy) not related to HIV infection or alcohol use were excluded.

Psychiatric illness and lifetime alcohol use were determined by experienced clinicians who administered the Structured Clinical Interview for DSM-IV-TR (SCID) to all participants [44]. Exclusion criteria were presence of dependence or abuse of substances other than alcohol or cannabis that exceeded length of alcoholism history or were more recent than alcoholism at study entry. Current nicotine dependence was not exclusion in this study. Participants in the alcohol groups met lifetime DSM-IV-TR criteria for alcohol dependence (one participant met criteria for alcohol abuse). Alcohol consumption over the lifetime and periods of heavy drinking (>6 drinks for men and >4 for women per day) were assessed by structured clinician-administered interview [45–47]. Current depression symptomatology was assessed using the Beck Depression Inventory-II [48]. Historical trauma information was collected by experienced clinicians through in-person interview (see [49]). Childhood trauma was defined in this study as a positive endorsement of experiencing > 1 of 13 Life Traumas before the age of 18 [49]. Table 1 summarizes the types of traumas that were reported to have been experienced during childhood. Thus, for the current analysis, all participants who met study eligibility criteria and had completed a SCID and trauma interview were included.

### Neuropsychological Measures

Participants eligible by medical and psychiatric assessment completed a cognitive test battery. The Dementia Rating Scale-2 assessed overall current general cognition, and the Wechsler Test of Adult Reading yielded an estimate of premorbid intelligence [50,51]. Handedness was determined using the Edinburgh Handedness Inventory or the Crovitz-Zener Handedness Questionnaire [52,53]. The battery (see [54]) comprised the following tests:

**Fine Finger Movement Test (n = 75)** measured simple motor speed and manual dexterity represented by the total number of turns of a knurled dowel made by each hand in 30 seconds. Group means for dominant and non-dominant hands separately were obtained [55].

**Finger Tapping (n = 77)** measured manual fine motor function by requiring participants to depress a telegraph key with their forefinger. Group means were obtained for three conditions: finger tapping with the forefinger of the dominant hand, the non-dominant hand, and alternating forefingers. Each mean score comprised average number of taps from three 15-second trials [56].

**Digit Symbol Test (n = 81)**, a subtest of the Wechsler Adult Intelligence Scale-Revised measured information processing speed, attention, visual scanning, and associative learning. The traditional raw score, i.e. the number of boxes correctly completed in 90 seconds via standard administration, was used [57].

**Trail Making Test Part A (Trails A) (n = 84)** assessed psychomotor speed, attention, sequencing, and visual scanning efficiency; participants connected numbered circles sequentially from 1 to 25 [58].

**Trail Making Test Part B (Trails B) (n = 81)** required participants to connect circles, sequentially alternating numbers and letters in order, which additionally assessed set-shifting ability.

**Stroop Color and Word Test (n = 81)** In the Word Reading condition, participants read color words (red, green, blue) from a list printed in black ink. In the Color Naming condition, they named the ink color of X’s printed in red, green, or blue. In the Color-Word condition (interference condition), participants named the color of the ink (red, green, or blue) that color words were printed in; however, the ink color and color word did not match, e.g., the word blue was printed in green ink. The scores for each condition were the number of items completed in 45 seconds [59,60].

**The Controlled Oral Word Association Test (F-A-S Test) (n = 84)** measured verbal fluency. Participants were given 60 seconds to list aloud words beginning with F, then A, and then S. The total number of words produced from each of the three trials was calculated [61].

**The Rey-Osterrieth Complex Figure Test (n = 84)** assessed immediate and delayed visuospatial episodic memory. Participants were asked to reproduce a complex line drawing (Copy Condition) [62,63]. Incidental recall of the figure was tested immediately after copy (Immediate Recall Condition) and again after a 30-minute delay (Delayed Recall Condition). Accuracy of the figure drawn at each condition was scored on a 36-point scale [64,65]. The immediate recall and delayed recall scores were expressed as a ratio of recall to copy score, thus representing a percentage of elements recalled as a function of copy to differentiate the memory components from the visuospatial component of this task.

**Logical Memory I (Immediate Recall) and II (Delayed Recall) Subtests of the Wechsler Memory Scales-Revised (WMS-R) (n = 72)** measured immediate and delayed verbal episodic memory [57]. Participants were asked to repeat two separate short narratives from memory. Logical Memory I comprised the sum of the correct elements recalled from each story immediately after hearing each story. Logical Memory II was the sum of correct story elements recounted after a 30-minute delay.

### Data Analysis

For demographic data analyses, chi-square tests and Fisher’s exact tests compared proportions among groups. Group comparisons of continuous data were conducted with one-way ANOVAs or Student’s t-tests, and group comparisons of data with limited ranges or non-normal distributions were analyzed with Kruskal-Wallis tests or Mann-Whitney U tests.

**Figure 1:**
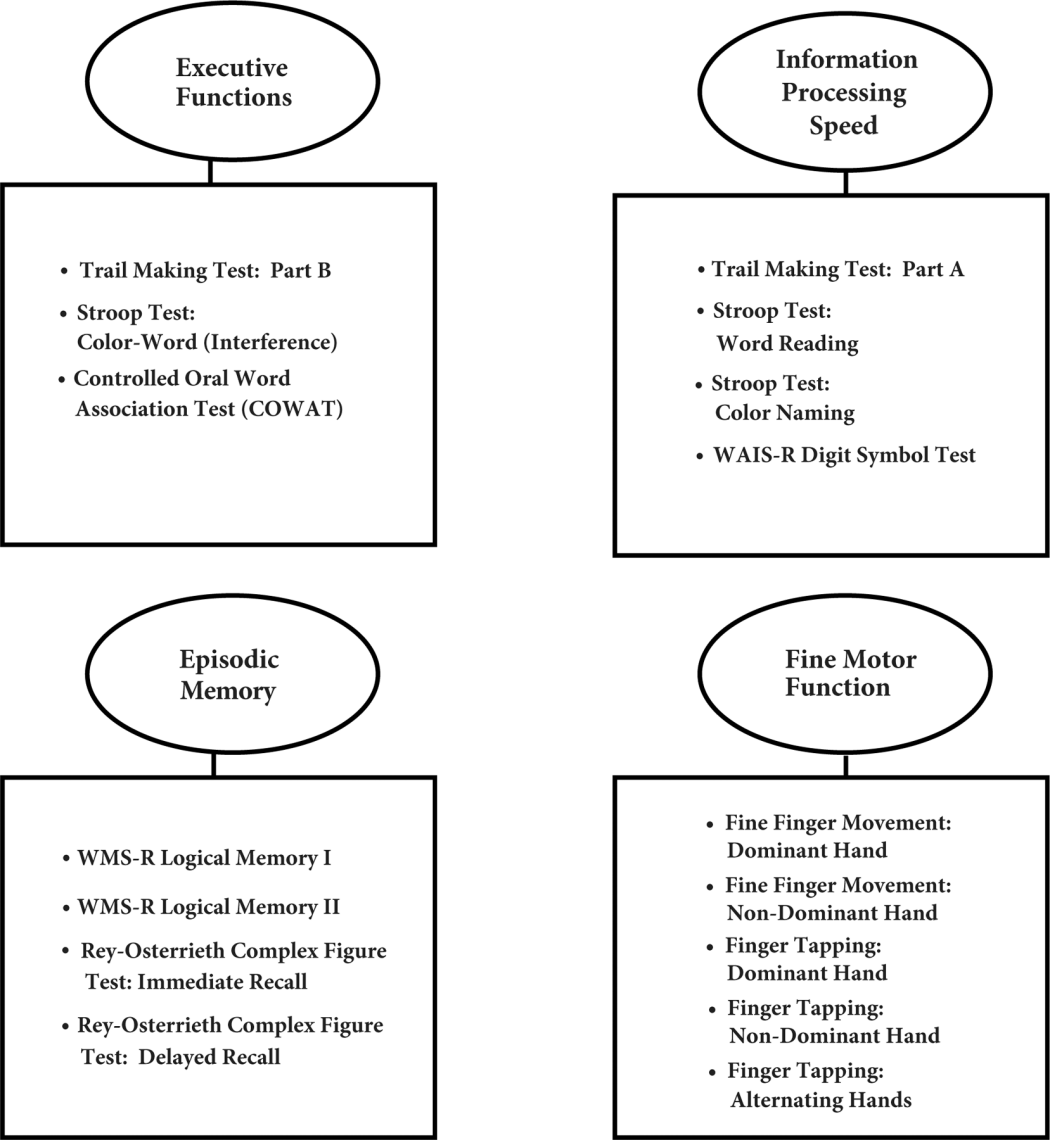
Four cognitive and motor domains examined (ovals) and the neuropsychological tests (squares) comprising each domain

To examine what underlying cognitive constructs may be related to interactive and independent contributions of alcoholism and trauma history in HIV infection, domain scores were examined first, as a method of data reduction. A second tier of analyses queried the individual tests underlying the domains to determine which specific tests contributed to the overall domain results. Z-scores for selective tests measuring related cognitive constructs were computed based on the performance of the comparison group to allow comparison of tests of varying scales of measurement and were combined to form four domain scores: executive function, information processing speed, episodic memory, and fine motor function domains. Thus, each participant’s domain scores were based on the mean of all available tests comprising that particular domain for that participant. For Rey-Osterrieth Immediate and Delayed Recall performance within the episodic memory domain, recall raw scores were adjusted for copy performance and thus each participant’s adjusted recall scores reflected a percentage of what was recalled based on what was copied. Directionality of cognitive constructs in analyses using Z-scores was adjusted as necessary so that lower Z-scores in every construct represented poorer performance. The neuropsychological tests comprising each of the four domains are shown in Figure 1.

To examine the interactions and main effects of childhood trauma and alcoholism status on the four domains of cognitive performance, 2×2 (alcohol dependence history, yes/no X childhood trauma history, yes/no) ANCOVAs, followed by Bonferroni post hoc tests corrected for multiple comparisons, were conducted. Age and education were entered as covariates in the ANCOVAs as these variables showed significant relationships with cognitive performance. Following initial analysis of the four domains of cognitive performance, constructs within each domain were examined with 2×2 ANCOVAs, controlling for age and education, to determine their specific relation with their corresponding domain results.

We sought to determine which clinical variables were interrelated and which disease- and trauma- related variables were related to cognitive performance. Pearson product-moment correlations queried relations between clinical variables, disease- and trauma-related variables and cognitive performance. All statistical analyses were conducted with PASW Statistics 18 and statistical significance was set at p< .05.

#### Demographic and Clinical Characteristics of the Participant Groups

Demographic characteristics of the four participant groups are presented in Table 2.

The four groups (H+Alc, H+Tr, H+Alc+Tr, and HComp) were comparable in all background demographics except ethnicity and Hepatitis C status. H+Alc and H+Alc+Tr had significantly more non-Caucasian participants than H+Tr, χ^2^ (3) = 11.88, p = .008. While the proportion of participants with Hepatitis C infection was less than 50% in all groups, H+Tr only had one person with Hepatitis C infection, a proportion significantly smaller than H+Alc+Tr, H+Alc, and HComp, χ^2^ (3) = 9.70, p = .02. Mean estimated premorbid verbal IQ as measured by the WTAR was similar among groups; no participants performed in the range for dementia on the Dementia Rating Scale-2.

The four groups were comparable in all HIV-related variables. The mean age of onset of HIV infection for all groups was reportedly in the 30s, and in all groups, participants reported that they had been living with HIV infection for a mean of approximately 20 years. Mean CD4 cell counts were >600 cells per mm^3^ and mean log viral load was < 2.0 copies for all groups.

**Figure 2:**
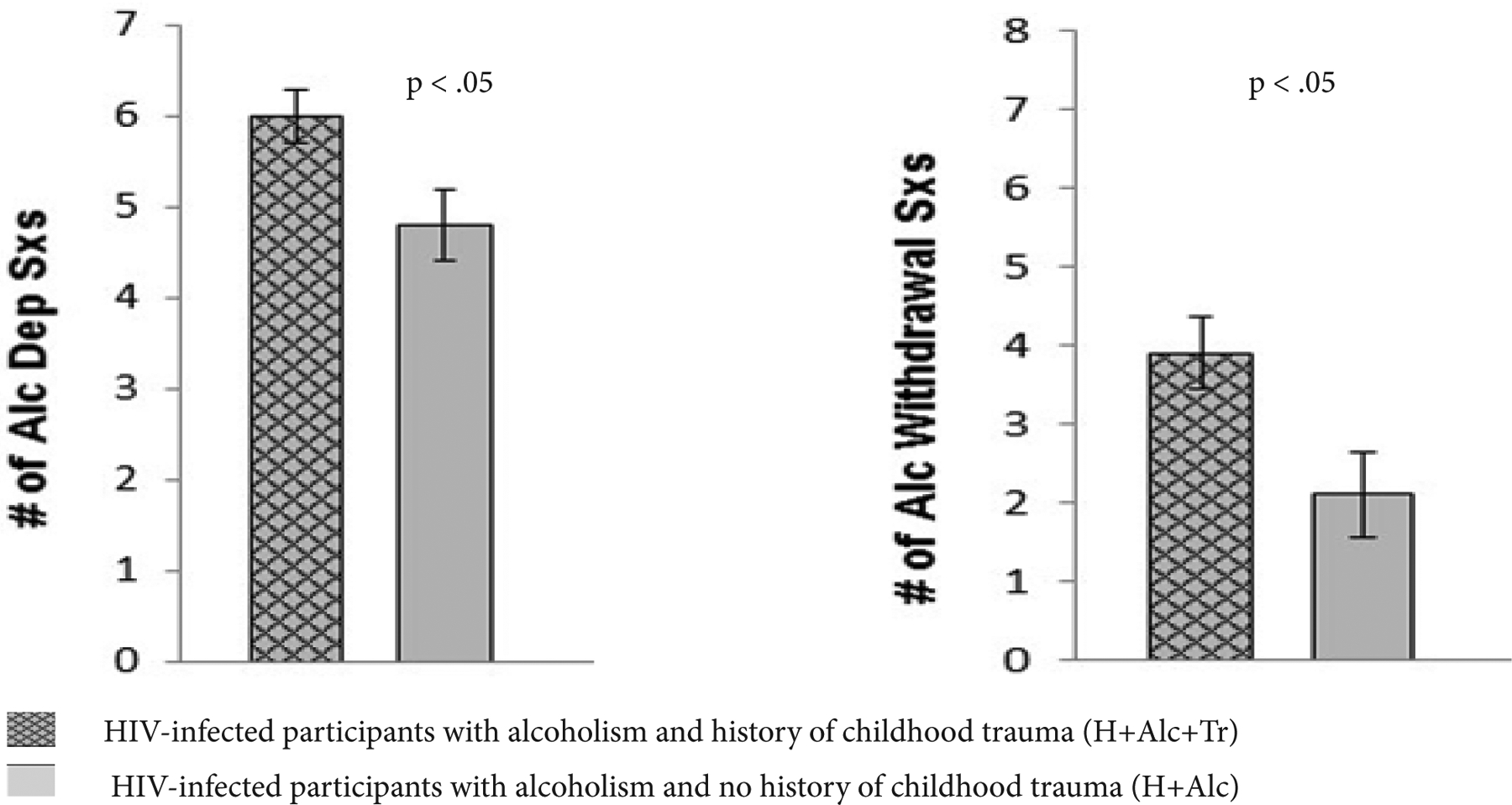
Mean number of alcohol dependence and alcohol withdrawal symptoms in HIV-infected alcoholic participants with and without history of childhood trauma

Although half to three-quarters of the participants in the four groups had a history of non-alcohol substance abuse, the median length of drug remission was at least 4 years for those with drug abuse history. H+Alc+Tr (57.1%) had the highest proportion of those with current nicotine dependence compared with H+Alc (42.1%), H+Tr (21.1%), and HComp (24.0%), χ^2^ (3) = 7.81, p = .05. Alcoholism-related demographic variables were comparable in the two alcohol groups, H+Alc+Tr and H+Alc. The mean age of alcoholism onset in alcohol groups was not statistically different: 26.8 years old in H+Alc+Tr and 20.4 years old in H+Alc. For H+Alc+Tr and H+Alc, the median length of alcohol dependence remission was at least 2.5 years, and drug remission was more remote than alcohol dependence remission by at least 1.5 years. Despite length of alcohol dependence remission, H+Alc+Tr and H+Alc were not abstinent from alcohol, as noted by a median of approximately 1 week since the date of last drink (sobriety). The alcohol dependent groups drank substantial volumes of alcohol over their lifetime (H+Alc+Tr: median = 727 kg; H+Alc = 1012 kg), which were at least 10 times greater than the lifetime volumes consumed by H+Tr (median = 71 kg) or HComp (median = 40 kg). H+Alc+Tr endorsed experiencing significantly more alcohol dependence criteria, t(38) = −2.52, p =.02, and alcohol withdrawal criteria, t(38) = −2.56, p =.02, than H+Alc (see Figure 2).

The mean age of onset of childhood trauma was 10.8 years old in H+Alc+Tr and 8.5 years old in H+Tr. In H+Alc +Tr, trauma was experienced prior to onset of alcoholism in all cases (see Table 2). Proportions of participants who reported experiencing more than one type of trauma included 57% of H+Alc+Tr and 42% of H+Tr. A high proportion of participants in the trauma groups reported experiencing physical, sexual, and/or emotional abuse during childhood (H+Alc+Tr: 66.7% and H+Tr: 89.5%). However, only 1 H+Tr and 5 H+Alc+Tr participants met DSM-IV-TR criteria for a diagnosis of PTSD in their lifetime. Incidence of a history of major depressive disorder was comparable among groups, at approximately 36% (p > .05), although a current major depressive episode was present in only 1 participant in each of the four groups. Mean BDI-II scores reflected minimal to mild current depressive symptomatology in each of the groups. Those in the trauma groups (H+Alc+Tr: 47.6% and H+Tr: 63.2%) were more likely to be currently taking an anxiolytic or antidepressant than those without history of trauma (H+Alc: 16.7% and HComp: 32.0%), χ^2^ (3) = 9.52, p = .03.

## Results

### Analyses of Domain Scores

**Figure 3:**
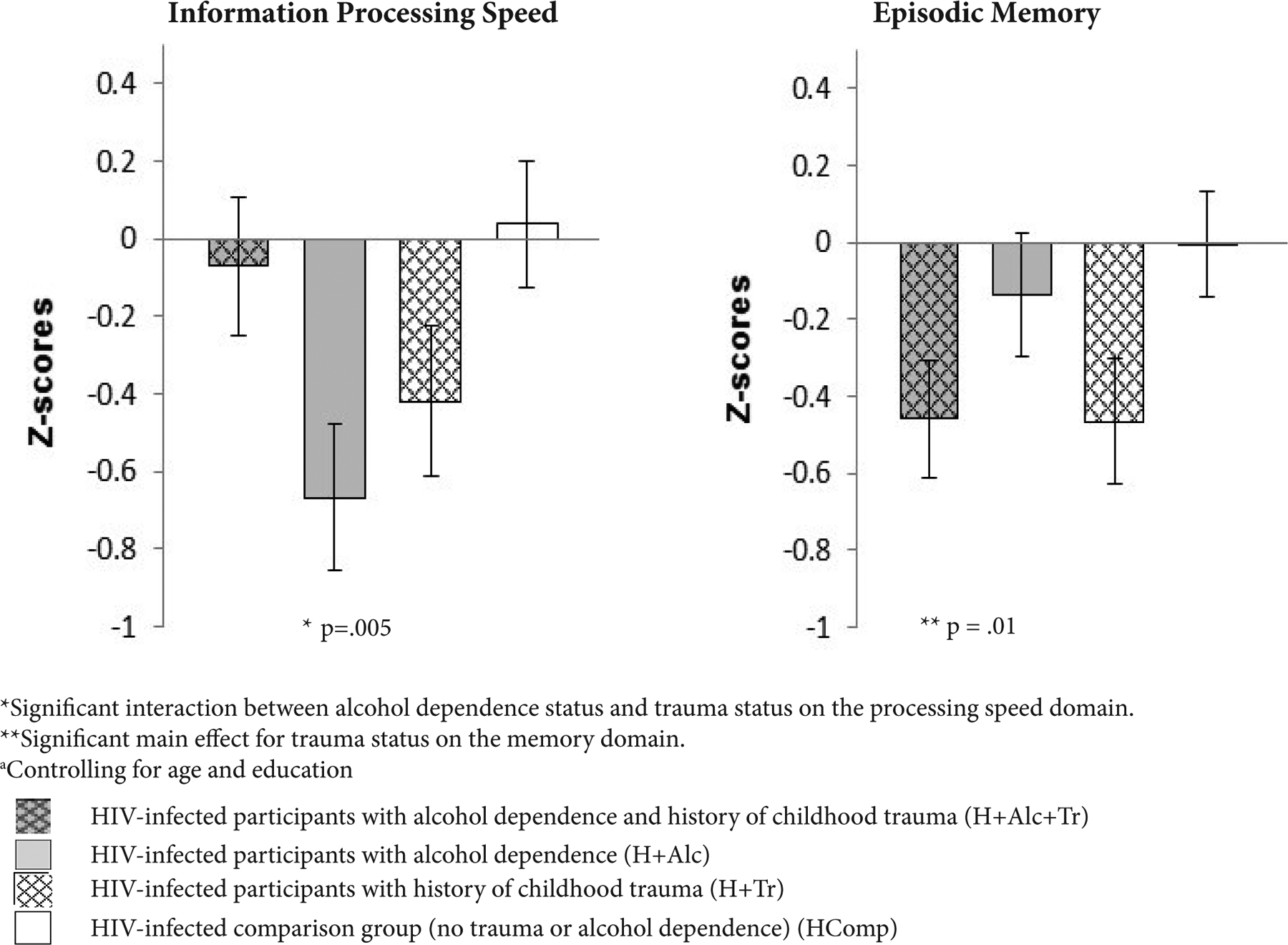
Z-score means and standard errors from significant ANCOVA analyses^a^ testing the interaction of alcohol dependence and childhood trauma history in HIV infection on cognitive domains

ANCOVAs, with age and education as covariates, tested the effects of childhood trauma status (history of childhood trauma, yes vs. no) and alcohol dependence status (history of alcohol dependence, yes vs. no) in HIV infection on four cognitive and motor function domains, Executive Functions, Information Processing Speed, Episodic Memory and Fine Motor Function. Significant differences among groups were found for Episodic Memory and for Processing Speed. Results are presented in Figure 3.

A main effect of trauma in HIV infection was found for the memory domain scores, F(1, 78) = 6.46, p = .01. Irrespective of alcoholism, participants with history of childhood trauma (H+Alc+Tr and H+Tr) had poorer episodic memory domain scores than those without history of childhood trauma (H+Alc and HComp).

An interaction between alcoholism and childhood trauma status in HIV infection for the Processing Speed domain scores, F(1, 78) = 8.44, p = .005, indicated that HIV-infected participants with alcoholism (H+Alc) had the slowest processing speed, which was significantly slower than that of the comparison group (HComp) (p = .04). HIV-infected participants with history of both alcoholism and childhood trauma (H+Alc+Tr) performed similarly to the comparison group while HIV-infected participants with childhood trauma (H+Tr) performed comparably to H+Alc.

Contrary to expectation, we did not see an interaction of childhood trauma status and alcohol dependence status on the fine motor skills domain scores. Moreover, the interaction between childhood trauma status and alcohol dependence status on the executive functioning domain scores was not significant. No significant main effects of trauma or of alcohol on the motor speed or executive function domain scores were found. Results can be found in Appendix 1.

### Group Comparisons of Individual Tests Comprising the Episodic Memory and Information Processing Speed Domains

Analysis of the cognitive tests in the episodic memory domain indicated that history of childhood trauma was significantly related to both poorer verbal recall and poorer visuospatial recall scores. On the immediate recall subtest of the WMS-R (Logical Memory I) (Table 3), both groups with childhood trauma history recalled significantly fewer story elements than the groups without childhood trauma, F(1, 66) = 12.69, p = .001. Similar results for verbal recall among those with childhood trauma compared to those without childhood trauma were found on the delayed recall subtest (Logical Memory II), F(1, 66) = 12.80, p = .001. After controlling for copy performance, groups with history of childhood trauma recalled significantly fewer figural elements on the immediate recall condition of the Rey-Osterrieth Complex Figure Test, F(1, 78) = 4.87, p = .04.

ANCOVA analyses controlling for age and education yielded significant interactions for childhood trauma status and alcohol dependence status for three of the four tests in the information processing speed domain: Trails A, Stroop Word Reading, and Stroop Color Naming (Table 3). Results of the analyses of these underlying tests mirrored those of the ANCOVA analysis of the domain scores. HIV-infected participants with childhood trauma and alcohol dependence (H+Alc+Tr) completed Trails A faster than HIV-infected participants with either alcohol dependence only (H+Alc) or childhood trauma only (H+Tr), F(1, 78) = 4.72, p = .03; however, their speed was comparable to that of the HIV-infected comparison group without alcoholism or trauma history (HComp). Similarly, H+Alc+Tr performed the Word Reading, F(1, 75) = 7.24, p = .01, and Color Naming, F(1, 75) = 4.97, p = .03, conditions of the Stroop Color and Word Test better than either H+Alc or H+Tr, and their performance was not significantly different from that of HComp.

Reanalysis of cognitive performance after removing the six participants with PTSD did not significantly change the results.

Results of the component tests of the Executive Functions and Motor Function domains can be found in Appendix 2; however, because no significant interactions or main effects of alcoholism status and childhood trauma status were found, no interpretation of these results are made here.

#### Relations among Clinical Variables and Domain Scores

Examination of relations among clinical variables within groups revealed that in those with a history of childhood trauma, a higher current log viral load was associated with a higher current BDI-II score, r = .38, p = .04. Among those with a history of alcoholism, greater quantity of alcohol consumed over the lifetime was associated with a higher current log viral load, r = .49, p = .005.

By examination of clinical variables and domain scores, we found that among those with a history of alcoholism, a greater number of alcohol dependence symptoms was associated with poorer memory domain scores, r = −.32, p = .05. There were no relations of HIV infection variables with domain scores among either trauma or alcoholism groups, nor were there any significant relations of trauma variables with domain scores within groups reporting childhood trauma.

## Discussion

To our knowledge, this is the first study to examine the relations of childhood trauma and alcoholism to cognitive functioning in HIV infection, all conditions known to have neurocognitive sequelae. We found that cognitive functioning in selective domains such as episodic memory and information processing speed was related to a history of childhood trauma and a lifetime alcoholism history. Our results, however, did not support the hypothesis of a compounded detrimental effect of childhood trauma and alcoholism in HIV infection on these domains.

Our sample comprised relatively healthy participants with HIV infection stratified into four groups: those with a history of alcohol dependence and childhood trauma (H+Alc+Tr), those with alcohol dependence (H+Alc), those with childhood trauma (H+Tr), and a comparison group without alcohol dependence or childhood trauma (HComp). HIV infection-related demographics were similar among groups. Participants acquired HIV infection in their 30s, were living with the infection for about 20 years, and almost all were on HAART medications. In alcohol groups, average age of onset of alcohol dependence was in the 20s, and more than 50% of alcoholics drank over 700 kg of alcohol in their lifetime. Length of sobriety was variable, with 50% of alcoholics reportedly consuming alcohol within the past week of testing, while others had remote sobriety of > 2 years. In those with a reported childhood trauma history, in every case, trauma history preceded alcoholism and HIV infection onset. The trauma reported included high proportion of abuse (physical, sexual, emotional); however, current depression levels and incidence of PTSD in this sample was relatively low.

Our results revealed an independent effect of childhood trauma status on episodic memory. HIV-infected individuals with a history of childhood trauma, irrespective of alcoholism history, exhibited poorer recall than HIV-infected individuals without childhood trauma history. These results are consistent with findings of a relation between early childhood adversity and memory deficits notably seen among PTSD samples [9–12,66], and now extend them to HIV-infected individuals with childhood trauma history, with and without alcoholism. Moreover, we found that both verbal memory and visual memory were affected in HIV-infected individuals with history of childhood trauma. Specifically, those with trauma evidenced poorer immediate and delayed verbal recall and poorer immediate visual recall than those without trauma history. Verbal memory appeared noticeably more affected than visual memory in the groups with childhood trauma history. One potential explanation may be the difference in the emotional content of materials used in the cognitive tasks assessing the memory domain. In the present study, visuospatial episodic memory evaluation requires the recall of a complex line drawing without any emotional component, while verbal episodic memory assessment involves a verbal recall test which may not be considered emotionally neutral. The WMS-R stories used in verbal memory testing involved a woman who was robbed of money and a truck driver who was in a road accident. Therefore, future research would appear essential to support and extend our findings regarding the episodic memory domain using verbal and visuospatial memory tasks with and without emotional salience. Further, our results may not necessarily be due to current level of depressive symptoms. Although trauma groups were more likely to be taking anxiolytics or antidepressants, current depressive episode incidence and level of depressive symptomatology were similar among groups. It also did not appear that a history of childhood trauma was related to poorer retention of information, as there was no significant difference between immediate and delayed recall scores for both verbal and visual episodic memory tests, a finding which concurs with that found in veterans with PTSD [10,67].

It remains unclear whether memory deficits associated with childhood trauma are sequelae from biological changes/stress due to the trauma or whether they are suggestive of a vulnerability to trauma. Smaller hippocampi in monozygotic twin pairs were shown to be a predisposing risk factor for a pathological response to combat stress [68]. It also may be that the trauma groups may not be benefitting from the influence of attention (executive skills) on memory, a relationship shown to be positively associated in the comparison group (HComp) and the alcoholic group (H+Alc) but not in the groups with history of childhood trauma (H+Tr and H+Alc+Tr).

We found an interactive effect of alcoholism and trauma status on information processing speed in HIV infection not in the direction expected. HIV-infected participants with alcohol dependence and childhood trauma history outperformed their counterparts with only alcoholism or only trauma, performing at a similar pace to the comparison group without alcoholism or childhood trauma. Thus, processing speed was faster in those with this “trimorbidity” compared to those with alcoholism but not childhood trauma despite a more severe clinical profile of alcohol dependence (greater number of DSM-IV alcohol dependence symptoms and withdrawal symptoms) in the trimorbid group. Further, greater impulsivity and hyperarousal may be factors in processing speed in HIV-infected individuals with a history of alcohol dependence and childhood trauma. These results are consistent with another study reporting that child physical abuse was related to impulsivity, particularly with respect to later diagnoses of attention-deficit hyperactivity disorder or bipolar disorder in adulthood [69].

Nevertheless, results of this study should be considered preliminary as this is first study of this kind and there are several limitations warranting consideration. First, the sample size is relatively small and used a retrospective design to query trauma history, which relies upon subject recall that is open to bias. We attempted to offset bias in recall by querying about particularly salient life traumas, e.g., abuse, life threatening events, using open-ended interview cuing regarding other life events to enhance memory, and examining trauma as a dichotomous yes/no variable. We did not address severity of trauma or chronicity of trauma. Another consideration is that many alcoholics were in remission, and thus the effects of alcoholism may not have been prominent; however, this may have benefited recollection of traumatic events, and moreover, most of the sample, while not drinking heavily, was not abstinent. It is also important to note that while a contribution of this study is the elucidation of psychiatric risk factors for compromised cognitive performance in HIV infection, this study was conducted without comparison groups that were HIV negative.

In conclusion, we found that childhood trauma history, irrespective of alcohol dependence history, was associated with poorer verbal and visual memory in HIV infection, although not necessarily with retention. We also found an interaction of alcohol and childhood trauma history in HIV infection, whereby those with both alcohol dependence and childhood trauma history demonstrate quicker psychomotor speed than their HIV-infected counterparts with either alcohol dependence or childhood trauma history and performed at a similar level to those HIV-infected individuals without alcohol dependence or abuse history. The latter findings could suggest that greater impulsivity and hyperarousal may be factors in processing speed in patients with the “trimorbidity”.

A recent literature review highlighted the lack of trauma-informed treatment [70]. Our current findings underscore the importance of both preventing childhood trauma and reducing the risk of subsequent alcoholism and risky behavior which could lead to HIV infection. Impeding the cascade of trauma to alcoholism/risky behavior to HIV infection via prevention and education could reduce the risk of verbal and visual memory impairment. Findings also suggest that screening for childhood trauma in HIV infection be an integral part of assessment prior to psychiatric or psychological treatment; the association of childhood trauma history and poorer memory in HIV infection has implications for therapeutic consequences including treatment outcome, medication adherence, following instructions, and quality of life.

## Figures and Tables

**Table 1:** Number of subjects reporting types of childhood life traumas in either trauma group

Types of Childhood Trauma Endorsed	H+Alc+Tr (n = 21)	H+Tr (n = 19)
Lost home in natural disaster	0	1
Life threatening accident, injury, illness	1	2
Forced sexual intercourse	9	4
Forced sexual touching	7	6
Physical abuse by parent/guardian	3	9
Physical abuse by boyfriend/girlfriend	2	1
Physical abuse by someone else	5	1
Physically assaulted or mugged	4	3
Emotional abuse by parent/guardian	6	9
Threatened with weapon/shot at (no injury)	2	0
Injured with weapon or shot at	0	0
Chased by someone where thought could get hurt	7	1
Car crash where someone was killed/badly injured	1	0

**Table 2:** Group demographic and clinical data

Demographic Variables	H+Alc+Tr % or Mean ± SD	H+Alc % or Mean ± SD	H+Tr % or Mean ± SD	HComp % or Mean ± SD	*p value*
n =	21	19	19	25	
**BACKGROUND DEMOGRAPHICS**					
Sex (% Men)	42.9%	78.9%	73.7%	60.0%	n.s.
Ethnicity (% Non-Caucasian)	85.7%	84.2%	42.1%	60.0%	.008 [H+Alc+Tr = H+Alc > H+Tr]
Age	56.6 ± 5.1	56.9 ± 5.4	54.4 ± 5.8	56.8 ± 7.9	n.s.
Education (years)	13.0 ± 2.1	13.0 ± 2.3	14.4 ± 1.5	13.4 ± 2.5	n.s.
Lifetime History of Drug Abuse (%)	66.7%	78.9%	47.4%	48.0%	n.s.
Hepatitis C (%)	47.4%	46.7%	5.3%	34.8%	.02 [H+Alc+Tr = H+Alc = HComp > H+Tr]
Beck Depression Inventory-II (BDI-II) Score*	13.5 ± 8.3; 11	8.2 ± 7.9; 4	10.6 ± 6.6; 10	7.4 ± 7.3; 6	n.s.
Dementia Rating Scale-2 Total Score^a^	134.7 ± 4.4	135.5 ± 3.6	136.6 ± 5.4	137.4 ± 3.8	n.s.
Wechsler Test of Adult Reading (premorbid IQ)^a^	84.6 ± 17.9	90.3 ± 16.9	98.8 ± 15.5	92.5 ± 19.3	n.s.
**TRAUMA-RELATED DEMOGRAPHICS**					
Trauma Age of Onset	10.8 ± 4.5	--	8.5 ± 5.0	--	n.s.
Physically/Sexually/Emotionally Abused (%)	66.7%	--	89.5%	--	n.s.
PTSD Diagnosis (%)	23.8%	--	5.3%	--	n.s.
**ALCOHOLISM-RELATED DEMOGRAPHICS**					
Alcoholism Age of Onset	26.8 ± 13.1	20.4 ± 5.0	--	--	n.s.
Alcohol Sobriety (months)	12.1 ± 39.3; 0.2	25.2 ± 37.0; 0.2	--	--	n.s.
Lifetime Alcohol Consumption (kg)*	1177 ± 1165; 727	1050 ± 692; 1012	78 ± 64; 71	68 ± 80; 40	<.001 [H+Alc+Tr = H+Alc > H+Tr = HComp]
**HIV-RELATED DEMOGRAPHICS**					
HIV Approx. Age of Onset	36.2 ± 5.4	34.4 ± 9.2	33.7 ± 10.9	37.5 ± 8.5	n.s.
Length of time with HIV (years)	20.4 ± 4.7	22.5 ± 6.9	20.7 ± 8.3	19.3 ± 7.1	n.s.
Log Viral Load	1.8 ± 0.9	2.0 ± 0.9	1.4 ± 0.2	1.8 ± 1.0	n.s.
CD4 Count*	602 ± 317; 492	727 ± 367; 591	773 ± 315; 798	679 ± 279; 637	n.s.
AIDS Status (%)	71.5%	73.7%	38.9%	64.0%	n.s.
HAART Medications (%)	85.7%	94.7%	94.7%	96.0%	n.s.

* Mean ± SD; median

a controlling for age and education

**Table 3:** Results of ANCOVA^a^ analyses of neuropsychological tests comprising significant cognitive domains in HIV-infected participants: with alcoholism and childhood trauma (H+Alc+Tr), alcoholism (H+Alc), childhood trauma (H+Tr), or comparison group with HIV infection (HComp)

DOMAIN Cognitive Test [description]	Raw Score Z-score^b^	H+Alc+Tr Adj. Mean (SE) Adj. Mean (SE)	H+Alc Adj. Mean (SE) Adj. Mean (SE)	H+Tr Adj. Mean (SE) Adj. Mean (SE)	HComp Adj. Mean (SE) Adj. Mean (SE)	Tr X Alc p value	Tr Main Effect Alc Main Effect p value
**INFORMATION PROCESSING SPEED**							
Trails A	**Raw Score**	**33.43**	**43.45**	**39.90**	**34.42**	.03	.53
[completion time in seconds]		**(3.52)**	**(3.71)**	**(3.84)**	**(3.21)**		.73
	Z-score	0.143	−0.792	−0.461	0.051		
		(0.329)	(0.346)	(0.358)	(0.299)		
Stroop Word Reading	**Raw Score**	**90.27**	**79.89**	**80.57**	**89.16**	.009	.80
[number of words read]		**(3.41)**	**(3.78)**	**(3.79)**	**(3.10)**		.95
	Z-score	0.092	−0.492	−0.454	0.029		
		(0.192)	(0.212)	(0.213)	(0.174)		
Stroop Color Naming	**Raw Score**	**64.49**	**61.30**	**60.70**	**68.44**	.03	.36
[number of colors named]		**(2.37)**	**(2.63)**	**(2.63)**	**(2.16)**		.50
	Z-score	−0.250	−0.481	−0.525	0.038		
		(0.172)	(0.191)	(0.191)	(0.157)		
Digit Symbol	**Raw Score**	**40.73**	**41.51**	**40.82**	**44.98**	.51	.34
[number of boxes completed]		**(2.54)**	**(2.69)**	**(2.77)**	**(2.27)**		.50
	Z-score	−0.332	−0.266	−0.325	0.025		
		(0.214)	(0.226)	(0.232)	(0.190)		
**EPISODIC MEMORY**							
WMS-R Logical Memory I	**Raw Score**	**16.31**	**21.25**	**15.74**	**22.67**	**.55**	.001
[number of correct story elements recalled]		**(1.62)**	**(1.73)**	**(1.82)**	**(1.43)**		.80
	Z-score	−0.628	−0.136	−0.685	0.006		
		(0.161)	(0.172)	(0.182)	(0.142)		
WMS-R Logical Memory II	**Raw Score**	**11.74**	**16.93**	**11.14**	**19.08**	.45	.001
[number of correct story elements recalled]		**(1.78)**	**(1.91)**	**(2.01)**	**(1.57)**		.68
	Z-score	−0.720	−0.208	−0.779	0.004		
		(0.176)	(0.188)	(0.198)	(0.155)		
Rey-Osterrieth Figure – Immediate Recall	**Raw Score**	**41.05**	**46.17**	**40.79**	**51.40**	.48	.04
		**(3.79)**	**(3.99)**	**(4.13)**	**(3.45)**		.53
	Z-score	−0.438	−0.210	−0.449	0.021		
		(0.168)	(0.177)	(0.183)	(0.153)		
Rey-Osterrieth Figure – Delayed Recall	**Raw Score**	**41.71**	**46.40**	**39.72**	**46.29**	.82	.18
[% figure elements recalled adj. for copy score]		**(4.05)**	**(4.26)**	**(4.41)**	**(3.69)**		.80
	Z-score	−0.203	0.015	−0.296	0.010		
		(0.188)	(0.198)	(0.205)	(0.172)		

a controlling for age and education

b lower Z-scores indicate poorer performance

**Appendix 1:**
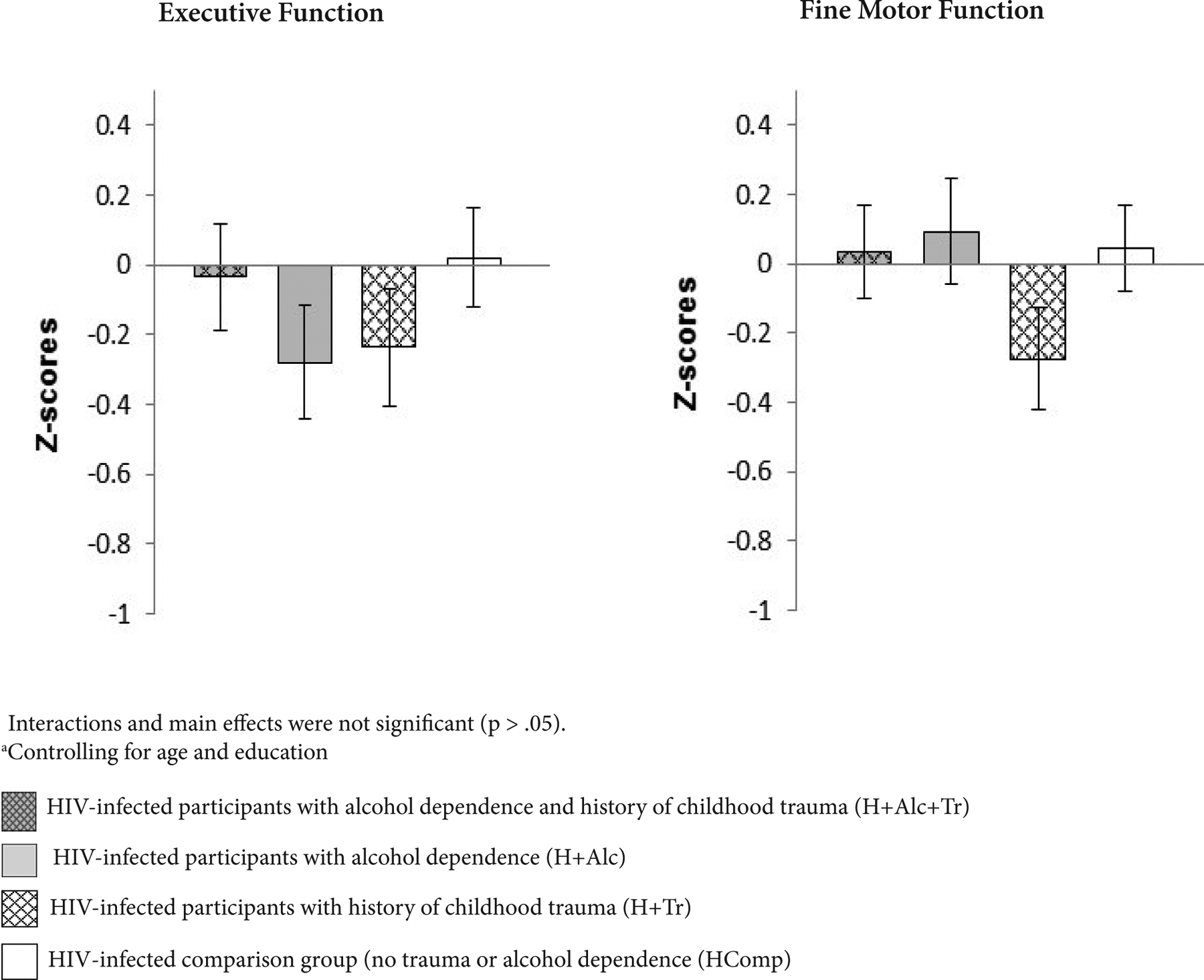
Z-score means and standard errors from non-significant ANCOVA analyses^a^ testing the interaction of alcohol dependence and childhood trauma history in HIV infection on cognitive and motor domains

## Appendix

**Appendix 2:** ANCOVA^a^ results for neuropsychological tests comprising non-significant cognitive and motor domains in HIV-infected participants: with alcoholism and childhood trauma (H+Alc+Tr), alcoholism (H+Alc), childhood trauma (H+Tr), or comparison group with HIV infection (HComp)

DOMAIN Cognitive Test [description]	Raw Score Z-score^b^	H+Alc+Tr Adj. Mean (SE) Adj. Mean (SE)	H+Alc Adj. Mean (SE) Adj. Mean (SE)	H+Tr Adj. Mean (SE) Adj. Mean (SE)	HComp Adj. Mean (SE) Adj. Mean (SE)	Tr X Alc p value	Tr Main Effect Alc Main Effect p value
**EXECUTIVE FUNCTION**							
Trails B	**Raw Score**	**98.77**	**137.75**	**113.72**	**95.02**	.01	.37
[completion time in seconds]		**(10.97)**	**(12.21)**	**(11.90)**	**(10.17)**		.24
	Z-score	−0.079	−0.930	−0.406	0.002		
		(0.239)	(0.266)	(0.260)	(0.222)		
Stroop Color-Word (Interference condition)	**Raw Score**	**32.61**	**36.28**	**33.72**	**37.46**	.99	.11
[number of colors named]		**(2.20)**	**(2.44)**	**(2.45)**	**(2.01)**		.62
	Z-score	−0.378	−0.073	−0.286	0.025		
		(0.183)	(0.203)	(0.203)	(0.167)		
Controlled Oral Word Association Test	**Raw Score**	**41.60**	**40.14**	**36.71**	**36.49**	.82	.76
[total number of words for F, A, S, combined]		**(2.69)**	**(2.84)**	**(2.94)**	**(2.46)**		.13
	Z-score	0.384	0.278	0.029	0.012		
		(0.196)	(0.206)	(0.213)	(0.178)		
**FINE MOTOR SKILLS**	**Raw Score**	**70.88**	**71.68**	**67.54**	**72.64**	.53	.39
Fine Finger Movement–dominant hand		**(3.40)**	**(3.70)**	**(3.49)**	**(2.93)**		.73
[number of turns of the dowel]	Z-score	−0.031	0.012	−0.207	0.062		
		(0.179)	(−0.195)	(0.184)	(0.155)		
Fine Finger Movement–non-dominant hand	**Raw Score**	**67.24**	**70.69**	**60.76**	**72.26**	.24	.03
[number of turns of the dowel]		**(3.41)**	**(3.71)**	**(3.50)**	**(2.94)**		.48
	Z-score	−0.194	−0.013	−0.533	0.069		
		(0.179)	(0.194)	(0.184)	(0.154)		
Alternated Finger Tapping–dominant hand	**Raw Score**	**57.31**	**62.84**	**53.55**	**56.98**	.65	.06
[number of key presses]		**(2.29)**	**(2.50)**	**(2.43)**	**(2.04)**		.04
	Z-score	0.078	0.577	−0.261	0.048		
		(0.207)	(0.226)	(0.219)	(0.184)		
Alternated Finger Tapping–non-dominant hand	**Raw Score**	**57.08**	**55.60**	**54.62**	**55.80**	.54	.95
[number of key presses]		**(2.14)**	**(2.33)**	**(2.27)**	**(1.90)**		.61
	Z-score	0.159	0.015	−0.080	0.035		
		(0.209)	(0.228)	(0.221)	(0.186)		
Alternated Finger Tapping – alternating hands	**Raw Score**	**64.97**	**61.16**	**58.35**	**63.77**	.20	.82
[number of key presses]		**(3.54)**	**(3.87)**	**(3.76)**	**(3.15)**		.58
	Z-score	0.110	−0.100	−0.255	0.044		
		(0.195)	(0.213)	(0.207)	(0.174)		

a controlling for age and education

b lower Z-scores indicate poorer performance
